# Bubble Test and Carotid Ultrasound to Guide Indication of Transesophageal Echocardiography in Young Patients With Stroke

**DOI:** 10.3389/fneur.2022.836609

**Published:** 2022-03-04

**Authors:** Ernst Mayerhofer, Dirk Kanz, Brigitte Guschlbauer, Christopher D. Anderson, Alexander Asmussen, Sebastian Grundmann, Christoph Strecker, Andreas Harloff

**Affiliations:** ^1^Department of Neurology and Neurophysiology, Faculty of Medicine, Medical Center—University of Freiburg, University of Freiburg, Freiburg, Germany; ^2^Department of Neurology, Massachusetts General Hospital, Harvard Medical School, Boston, MA, United States; ^3^Department of Neurology, Brigham and Women's Hospital, Boston, MA, United States; ^4^Department of Cardiology and Angiology I Heart Center, Faculty of Medicine, Medical Center—University of Freiburg, University of Freiburg, Freiburg, Germany

**Keywords:** ischemic stroke, transient ischemic attack, diagnostic imaging, ultrasound, bubble test method, carotid intima-media thickness, transesophageal echocardiography (TEE)

## Abstract

**Background and Purpose:**

Indication of transesophageal echocardiography (TEE) in patients ≤60 years with brain ischemia is uncertain.

**Methods:**

This prospective double-blinded study included patients with cryptogenic acute ischemic stroke or transient ischemic attack (TIA) ≥18 and ≤60 years. After routine diagnostics, all patients underwent patent foramen ovale (PFO) screening by transcranial Doppler (TCD) bubble test, carotid ultrasound for atherosclerosis screening (intima-media-thickness >0.90 mm or plaques), and TEE. We calculated sensitivity, specificity, positive predictive values (PPV), and negative predictive values (NPV) of the combined non-invasive ultrasound to predict therapy-relevant TEE findings.

**Results:**

We included 240 consecutive patients (median 51 years, 39% women) of which 68 (28.3%) had both a negative bubble test and no carotid atherosclerosis. Of these, 66 (97.1%) had unremarkable TEE findings; in one patient a small PFO was found and closed subsequently, in another patient a 4.9 mm thick aortic atheroma was found, and double platelet inhibition initiated. Of the other 172 (71.7%) patients, 93 (54%) had PFO and 9 (5.2%) complex aortic plaques. No other therapy-relevant findings were present in both groups. Non-invasive ultrasound had a sensitivity of 98.0%, specificity of 47.8%, NPV of 97.1%, and PPV of 58.1% for therapy-relevant TEE findings.

**Conclusions:**

Bubble test and carotid ultrasound could be used for the individual decision for/against TEE in patients with cryptogenic stroke ≤60 years. If they are unremarkable, TEE can be omitted with high safety regarding secondary prevention. If bubble test is positive and/or carotid ultrasound shows atherosclerosis, TEE should be carried out if PFO or aortic atheroma are potentially relevant for further patient management.

## Introduction

Detailed diagnostic work-up for the identification of stroke etiology is crucial to optimize secondary prevention and avoid recurrent brain ischemia. But even after comprehensive diagnostics, stroke etiology remains cryptogenic in 22–43% of patients (1–3). In such cases, transesophageal echocardiography (TEE) can be helpful to detect further cardiac or aortic sources of brain embolism (4–7).

Transesophageal echocardiography findings in patients with stroke differ significantly between age groups. Complex aortic plaques (i.e., ≥4 mm thick, ulcerated, or containing superimposed thrombi), left atrial appendage thrombi, and indirect signs of atrial fibrillation, such as spontaneous echo contrast or decreased left atrial appendage emptying velocity, are predominantly detected in older patients (8). In patients under 60 years, however, TEE shows mainly patent foramen ovale (PFO) and infrequently complex aortic plaques while other embolic sources are very rare (8, 9). Thus, the benefit of TEE in young patients with stroke lies in the detection of PFO and, in fewer cases, complex aortic plaques.

Patent foramen ovale closure reduces recurrence risk in patients ≤60 years of age with cryptogenic stroke with a potentially higher benefit in cases with moderate or high right-to-left shunt (RLS) or atrial septal aneurysm (10–13). Optimal treatment of complex aortic plaques is not yet evidence-based and patients are usually treated by oral anticoagulation or dual platelet inhibition for several weeks followed by single platelet inhibition (14–16). However, the detection of such plaques is also important in the context of determining stroke etiology and initiating intensified statin treatment (8). Transesophageal echocardiography has a favorable safety profile, but because of its invasive nature, a minimal risk remains for major complications, such as placement failure, bronchospasm, atrial fibrillation, and oropharyngeal bleeding; in rare cases it can lead to serious complications, such as esophagus perforation, mediastinitis, and death (17).

Remarkably, extra- and intracranial ultrasound can reliably exclude PFO and complex aortic plaques and could therefore guide the indication for TEE in young patients with stroke. Transcranial Doppler (TCD) bubble test can detect or rule out PFO with a sensitivity of 97% and specificity of 93% compared with TEE (18, 19). In contrast, the sensitivity of transthoracic echocardiography (TTE) in detecting PFO is only about 50% compared with TEE (19). The TCD Doppler bubble test is a quick, convenient, and safe bedside procedure with fully alert patients that are able to perform a proper Valsalva-maneuver to increase sensitivity for PFO detection (20–22). Furthermore, complex aortic plaques closely coincide with carotid atherosclerosis. Accordingly, carotid ultrasound can reliably exclude complex aortic plaques with a high negative predictive value (NPV) of 91–95% (23–26). These two non-invasive approaches are already established as part of the work-up in current guidelines (14, 27, 28). However, the overall diagnostic value of TEE after screening by the combination of these two ultrasound examinations especially in patients with cryptogenic stroke ≤60 years has not yet been investigated.

Therefore, the goal of our study was to prospectively investigate in young patients with stroke if (i) PFO and complex aortic atheroma are the only stroke-relevant TEE findings and (ii) those can be reliably predicted by established non-invasive ultrasound methods.

## Methods

### Study Cohort

This prospective clinical observational study was conducted in the Department of Neurology of our University Medical Center. Between August 2019 and April 2021, all consecutive patients presenting to the neurological intensive care and stroke unit ≥18 and ≤60 years with acute ischemic stroke, transient ischemic attack (TIA), amaurosis fugax, or retinal artery occlusion were screened daily. We included patients with cryptogenic etiology after routine diagnostic workup who underwent TEE. Performance of TEE was at the discretion of the treating physician team and indicated depending on characteristics and brain infarction pattern of patients (embolic vs. non-embolic). Routine diagnostics comprised of comprehensive assessment of medical history, physical examination, brain imaging with CT and/or MRI, vascular imaging of extra-and intracranial arteries by CT- or magnetic resonance (MR)-angiography or 2D carotid duplex sonography, 12-lead- and >24 h Holter- or monitor-ECG, routine laboratory tests, and TTE [we did not assess for PFO on TTE due to its low sensitivity (19)]. Supplementary tests, such as factor II and V mutations, antiphospholipid antibodies, complement factors, antinuclear antibodies, and antineutrophil cytoplasmic antibodies, or CSF analysis were carried out at the discretion of the treating physician. Patients were only included if routine diagnostics, such as TTE showed no conclusive source for brain ischemia. All patients underwent additional non-invasive ultrasound and TEE (see below). Patients with PFO were screened for deep vein thrombosis using the Wells score (29). Compression ultrasound of the pelvic and leg veins was performed in patients with high risk or clinical suspicion for thrombosis. In patients with low or moderate risk, it was performed in case of increased age-adjusted D-dimers (30, 31). Etiology of stroke was classified according to the modified Trial of Org 10172 in Acute Stroke Treatment (TOAST) criteria (32) by two experienced neurologists after completion of diagnostics, such as TEE. Cardiovascular risk factors were obtained from laboratory tests and the medical charts of patients and are described in detail in the Supplementary Methods. We calculated the Risk of Paradoxical Embolism (RoPE) score for patients with positive TCD bubble test and for those with PFO on TEE (33–36).

Exclusion criteria were determined as stroke etiology prior to TEE, suspicion of endocarditis, contraindications for TEE (e.g., severe thrombocytopenia, known esophageal pathologies with increased risk of esophageal injury, unstable clinical condition, and patient refusal), impossibility to perform bubble test (no transtemporal acoustic window, unable to carry out Valsalva-maneuver because not alert/intubated), or refusal to participate in the study.

### Non-Invasive Ultrasound

Transcranial Doppler bubble test and carotid ultrasound were performed on a Philips EPIQ 7 Elite ultrasound device (Koninklijke Philips N.V., Amsterdam, The Netherlands) equipped with software for the automatic delineation and measurement of the intima media complex [Philips Q-App Intima Media Thickness (IMT)]. We used an S5-1 sector array transducer for transcranial and an L12-3 linear array transducer for extracranial ultrasound. To minimize inter-observer variability, all study examinations were carried out by a team of three experienced examiners who completed a collective training on one patient prior to the start of the study and the first 10 examinations were performed by all three. Cardiologists performing TEE and neurologists performing non-invasive ultrasound were blinded to the results of each other. To ensure blinding, we scheduled the non-invasive ultrasound before TEE and shared the results with the patients, the physicians, and in the electronic patient chart only after the final publication of the TEE examination. Non-invasive ultrasound was only considered negative if both bubble test and screening for carotid atherosclerosis were negative.

#### Screening for PFO

**Figure 1:**
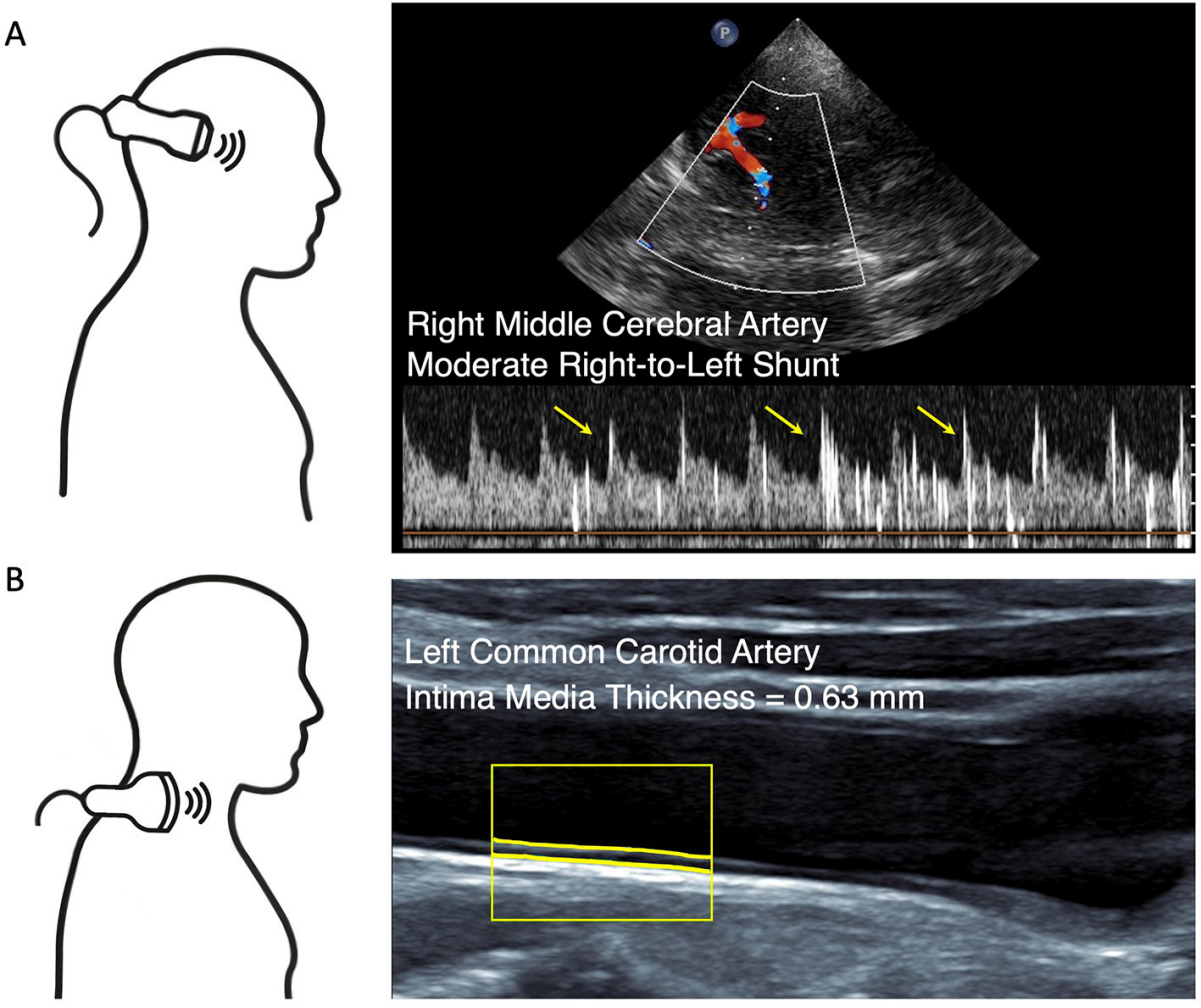
Non-invasive ultrasound. **(A)** Transcranial Doppler (TCD) bubble test at rest. Duplex-guided Doppler velocity spectrum of the right middle cerebral artery reveals moderate right-to-left shunt within 14 s after the injection of agitated saline. Yellow arrows point to exemplary high-intensity signals. **(B)** Carotid ultrasound with software delineation of the intima media complex reveals normal intima media thickness (IMT).

For non-invasive PFO detection or exclusion, we performed TCD bubble test in all patients at rest and while performing an active Valsalva maneuver. We used a slightly modified approach of the examination consensus to improve the test sensitivity by adding 1 ml of fresh patient blood to the agitated solution as suggested earlier (21, 37, 38). Quantification of RLS was based on the visual and audible detection of micro-embolic high-intensity signals on the Doppler blood flow-velocity spectrum of the right middle cerebral artery. Bubble test was considered positive if there was one or more high-intensity signals at rest or under Valsalva maneuver (Figure 1). The examination procedure is described in detail in the Supplementary Methods.

#### Screening for Carotid Atherosclerosis

Extracranial ultrasound was performed in a supine position on both sides of the neck. We measured mean IMT over a 10 mm distance at the transducer-distant side in a longitudinal section 20–30 mm proximal of the bifurcation at a plaque-free site of the common carotid artery. Furthermore, the distal common and the proximal internal and external carotid arteries were screened for the presence of atherosclerotic plaques (Figure 1). Plaques were defined according to the international consensus as circumscribed wall thickening >1.5 mm (39). Screening for carotid atherosclerosis was considered positive if the mean IMT on at least one side exceeded 0.90 mm [as previously suggested (23, 24)] or atherosclerotic plaques were present.

### Transesophageal Echocardiography

Transesophageal echocardiography examination in patients with stroke was recently described in detail (8) and performed in a standardized manner on a Philips Affiniti 70c or EPIQ 7 Cvx ultrasound device equipped with a X7-2t transducer. Patent foramen ovale was diagnosed by injection of agitated gelatin polysuccinate. Right-to-left shunt was categorized into small (<10 bubbles), moderate (10–20 bubbles), and large (>20 bubbles) shunts. Aortic plaques were detected and measured in a standardized manner and categorized according to previous studies (40, 41): no atherosclerosis, atherosclerosis without plaques (irregular thickening of the intima with increased echogenicity), atherosclerosis with plaques (plaques ≥2 and <4 mm thickness without ulceration or mobile components), and complex plaques (≥4 mm thick, ulcerated, or containing superimposed mobile thrombi). We screened the left atrium for the presence of spontaneous echo contrast and fixed or mobile echo-dense masses (i.e., thrombi). The left atrial appendage end-diastolic peak flow velocity was estimated by the mean value of five Doppler measurements in the proximal third of the left atrial appendage. Transesophageal echocardiography was performed by an experienced examiner and in addition, the recorded images/videos were reviewed by the supervising attending physician cardiologist. Cardiologists involved in TTE/TEE were blinded to the results of the non-invasive ultrasound (see above).

### Statistics

Incomplete datasets with regard to non-invasive ultrasound or TEE were excluded for analysis. Not normally distributed measures as assessed with the Shapiro-Wilk test are given as median and interquartile range (IQR), normally distributed measures are given as mean ± SD. We used Buderer's formula for sample size calculation taking into account the prevalence of disease, sensitivity, specificity, and precision (42). We estimated a needed sample of 182 patients with an expected sensitivity of 95%, desired precision of 5%, and expected prevalence of 40% for potential therapy-relevant findings on TEE (40% PFO, 5% complex aortic plaques, and 2% other; not mutually exclusive) (8). We used the qualitative non-invasive ultrasound results as index test and qualitative results of TEE (therapy-relevant findings) as reference test to calculate the sensitivity, specificity, positive predictive values (PPV), NPV, and positive and negative likelihood ratios. We calculated *CI*s for sensitivity, specificity, PPV, and NPV using exact Clopper–Pearson intervals, and for likelihood ratios using formulae by Simel et al. (43). All statistical analyses were performed in RStudio version 1.4.1106 with R version 4.1.0 using xlsx and epiR library (44).

## Results

### Study Cohort

**Figure 2:**
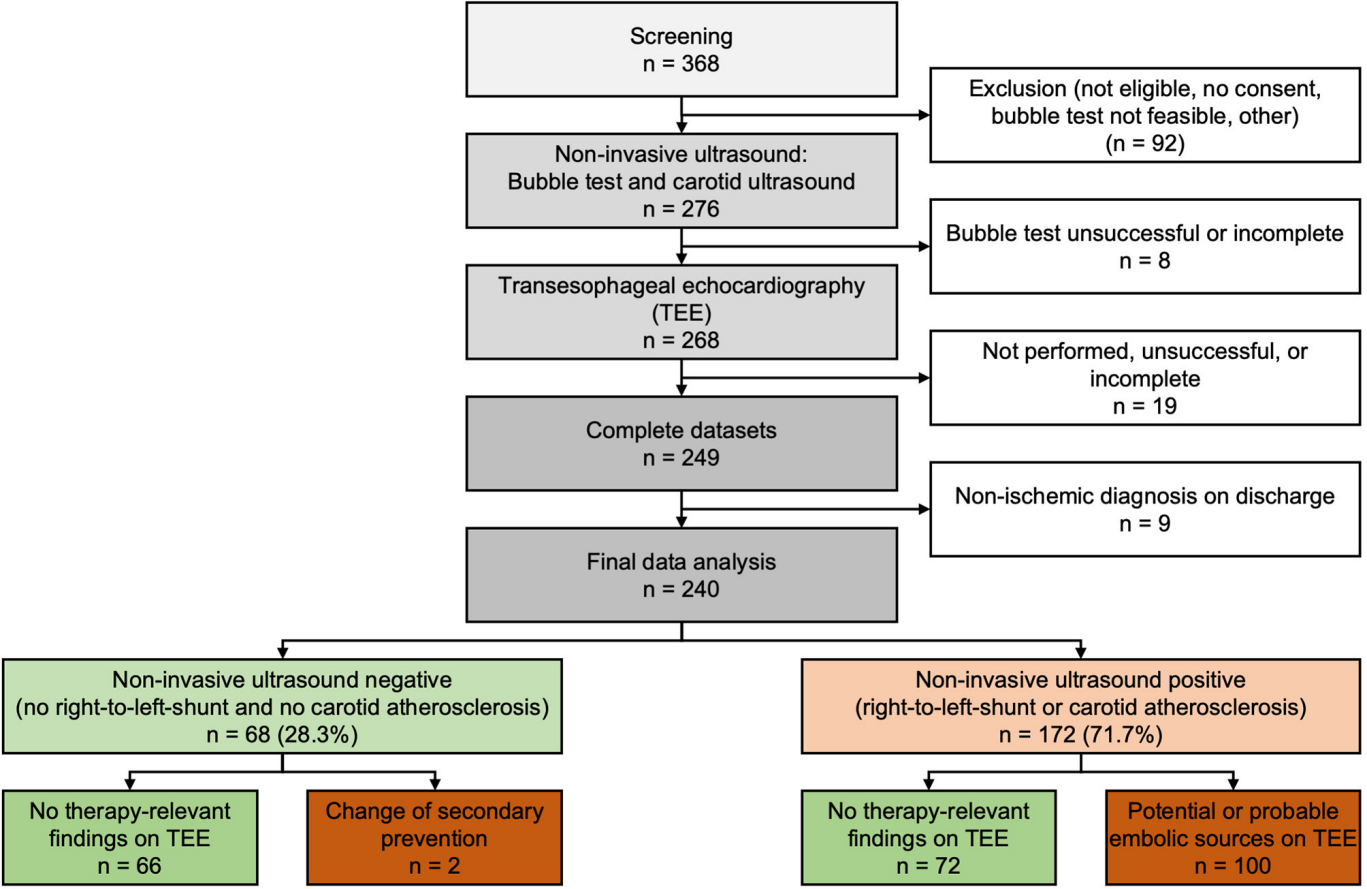
Recruitment and diagnostic results of patients in the study cohort.

We prospectively screened 368 consecutive patients and included 276 patients. Upon completion of the study, 27 patients were excluded because of incomplete non-invasive ultrasound or TEE data. To consider only patients with cerebral or retinal ischemia, we excluded nine further patients who underwent stroke diagnostics but had a non-ischemic diagnosis at discharge. Baseline characteristics of the 240 patients that had undergone complete non-invasive ultrasound and TEE and were finally included are given in Table 1. The study flowchart is displayed in Figure 2. Reasons for non-inclusion, exclusion, and missing data are given in the Supplementary Results. Transesophageal echocardiography was performed within 1 day in 203 (84.6%) and within 2 days of non-invasive ultrasound in 228 (95%) of the patients.

**Table 1:** Baseline characteristics of the 240 study participants.

**Characteristic**	**Value**
Female—*n* (%)	93 (38.8%)
Age, years^†^	51 (44–58)
Qualifying event—*n* (%)	
Ischemic stroke	163 (67.9%)
Transient ischemic attack	63 (26.3%)
Amaurosis fugax	13 (5.4%)
Retinal artery occlusion	1 (0.4%)
Etiology after complete diagnostic work-up including transesophageal echocardiography—*n* (%)	
Large-artery atherosclerosis	14 (5.8%)
Cardioembolism	72 (30%)
Patent foramen ovale	56 (23.3%)
Small-vessel disease	18 (7.5%)
Other determined etiology	13 (5.4%)
Undetermined etiology	123 (51.3%)
Two or more etiologies	1 (0.4%)
Cryptogenic	122 (50.8%)
Cardiovascular risk factors—*n* (%)	
Dyslipidemia	138 (57.5%)
Smoker	102 (42.5%)
Hypertension	81 (33.8%)
Obesity	57 (23.8%)
Family history of stroke or cardiovascular disease	56 (23.3%)
History of ischemic stroke or transient ischemic attack	27 (11.3%)
Diabetes mellitus	26 (10.8%)
Coronary artery disease	9 (3.8%)

† *Median and interquartile range (IQR)*.

### Non-Invasive Ultrasound

The bubble test was positive in a total of 119 patients (49.6%), with 80 patients (33.3%) showing a positive test at rest and 39 patients (16.3%) showing a positive test under Valsalva maneuver. Of those with positive bubble test, 38 (32%) had a small RLS, 37 (31%) had a moderate RLS, and 44 (37%) had a large RLS. The median RoPE score was 4 (IQR 3–5) and 74 (62%) of the patients with positive bubble test had a RoPE score of 4 or more. Carotid atherosclerosis was present in 102 patients (42.5%) due to increased IMT (*n* = 41, 17.1%) and/or due to plaques (*n* = 92, 38.3%). The detailed results of the non-invasive ultrasound diagnostics are given in Supplementary Table 1. For subsequent analyses, we divided the patients based on non-invasive ultrasound results in two groups. In total, 68 patients (28.3%) showed unremarkable non-invasive ultrasound, i.e., negative bubble test and absence of carotid atherosclerosis (Figure 2, left branch). In the remaining 172 patients (71.7%), we found either a positive bubble test or carotid atherosclerosis (Figure 2, right branch).

### Transesophageal Echocardiography

Of 68 patients, 66 patients with unremarkable non-invasive ultrasound showed no therapy-relevant TEE results (97.1%). Moreover, two of them had a PFO with small RLS, one had moderate RLS only under Valsalva maneuver, and another had an atrial septal defect (ASD) with no RLS. None of these patients underwent interventional closure of PFO/ASD. However, the remaining two patients showed therapy-relevant findings in TEE: in one patient with embolic infarction, TEE showed contrast agent flow from pulmonary veins suspicious for pulmonary arteriovenous malformation as well as a small PFO with small RLS at rest. Subsequent chest CT ruled out arteriovenous malformation but showed incidental bilateral pulmonary embolism ultimately leading to oral anticoagulation and recommendation of PFO occlusion. The other patient with TIA showed a 4.9 mm thick aortic plaque without ulceration or mobile components and was treated with dual platelet inhibition for 3 months (with single platelet inhibition thereafter) as an individual treatment recommendation.

In contrast, more than half of the patients (*n* = 100, 58.1%) with positive non-invasive ultrasound had either PFO (*n* = 93, 54.1%) or complex aortic plaques (*n* = 9, 5.2%). In patients with PFO, venous duplex imaging was performed in 54 cases (55.6% of patients with PFO) with evidence of deep venous thrombosis in six cases (6.5% of patients with PFO). The RoPE score in patients with PFO on TEE was similar to patients with positive bubble test: median 4 (IQR 3–5, *p* = 0.7) and 58 (60%) of the patients with PFO had a RoPE score of 4 or more.

No further therapy-relevant findings were obtained in both groups. Both groups included one patient with indirect signs of atrial fibrillation (left atrial appendage flow velocity <30 cm/s). However, prolonged and continuous monitor-ECG for 4 and 5 days, respectively, did not reveal atrial fibrillation. The detailed TEE results are given in Supplementary Table 2.

### Prediction of Therapy-Relevant Findings in TEE by Non-Invasive Ultrasound

Combined non-invasive ultrasound reached a sensitivity of 98.0% (95% *CI* [93.1, 99.8]) and an NPV of 97.1% (95% *CI* [89.8, 99.6]) for therapy-relevant findings in TEE. By contrast, specificity was 47.8% (95% *CI* [39.3, 56.5]) and PPV was 58.1% (95% *CI* [50.4, 65.6]). The positive likelihood ratio was 1.88 (95% *CI* [1.6, 2.2]) and the negative likelihood ratio was 0.04 (95% *CI* [0.01, 0.16]). Results of a cross tabulation of non-invasive ultrasound vs. TEE are given in Table 2.

**Table 2:** Cross tabulation of non-invasive ultrasound vs. transesophageal echocardiography (TEE).

	**Therapy-relevant findings on transesophageal echocardiography**		
**Non-invasive ultrasound**	**Yes**	**No**	**Sum**
**Positive**	100	72	172
**Negative**	2	66	68
**Sum**	102	138	240

We performed separate analyses for TCD bubble test and carotid atherosclerosis regarding the prediction of PFO and aortic plaques, respectively. Transcranial Doppler bubble test alone had a sensitivity of 93.8% (95% *CI* [86.9, 97.7]) and an NPV of 95.0% (95% *CI* [89.5, 98.2]) for detecting and excluding PFO in TEE. Specificity was 79.9% (95% *CI* [72.4, 86.1]) and PPV was 75.6% (95% *CI* [66.9, 83.0]). Carotid atherosclerosis alone showed a sensitivity of 90.0% (95% *CI* [55.5, 99.7]) and an NPV of 99.3% (95% *CI* [96.0, 100.0]) for the detection and exclusion of complex aortic plaques. Due to the overall low prevalence of complex aortic plaques (*n* = 10, 4.2%), specificity was 59.6% (95% *CI* [52.9, 66.0]) and PPV was only 8.8% (95% *CI* [4.1, 16.1]).

To confirm robustness of the results, we performed sensitivity analyses in the subset of patients with cryptogenic stroke only (*n* = 163, 67.9%). The values obtained were very similar to those in the whole cohort and are given in the Supplementary Results.

## Discussion

This prospective double-blinded diagnostic accuracy study in patients up to 60 years with cryptogenic brain or retinal ischemia (i) confirmed that TEE findings are mainly PFO and aortic atheroma and (ii) showed that transcranial bubble test and carotid ultrasound can serve as an easy and valuable diagnostic tool for the decision for/against TEE.

We could confirm that TEE in young patients with stroke predominantly detects PFO (ca. 40%), a few complex aortic plaques, and only very rarely other findings, such as indirect signs of atrial fibrillation. Furthermore, we established a diagnostic pre-test to reliably exclude patients in which TEE will have a very low chance to yield a relevant finding: in 28% of all 240 patients, non-invasive ultrasound was unremarkable (i.e., bubble test was negative and carotid atherosclerosis was absent). The risk of missing a therapy-relevant finding in these patients by omitting TEE was very low: only one small PFO and one 4.9 mm thick aortic plaque without superimposed thrombus would have been missed by our approach, and no high-risk source of cerebral embolism would have been overlooked. With a sensitivity of 98.0% and an NPV of 97.1%, TEE could have been omitted without the risk to overlook relevant sources of stroke to save resources and to furthermore increase safety and comfort in these patients. By contrast, the 72% of patients with positive non-invasive ultrasound had a more than 50% chance that PFO or aortic atheroma would be detected on TEE. In patients who can benefit therapeutically from those findings, TEE can aid in determining stroke etiology and improving secondary prevention.

**Figure 3:**
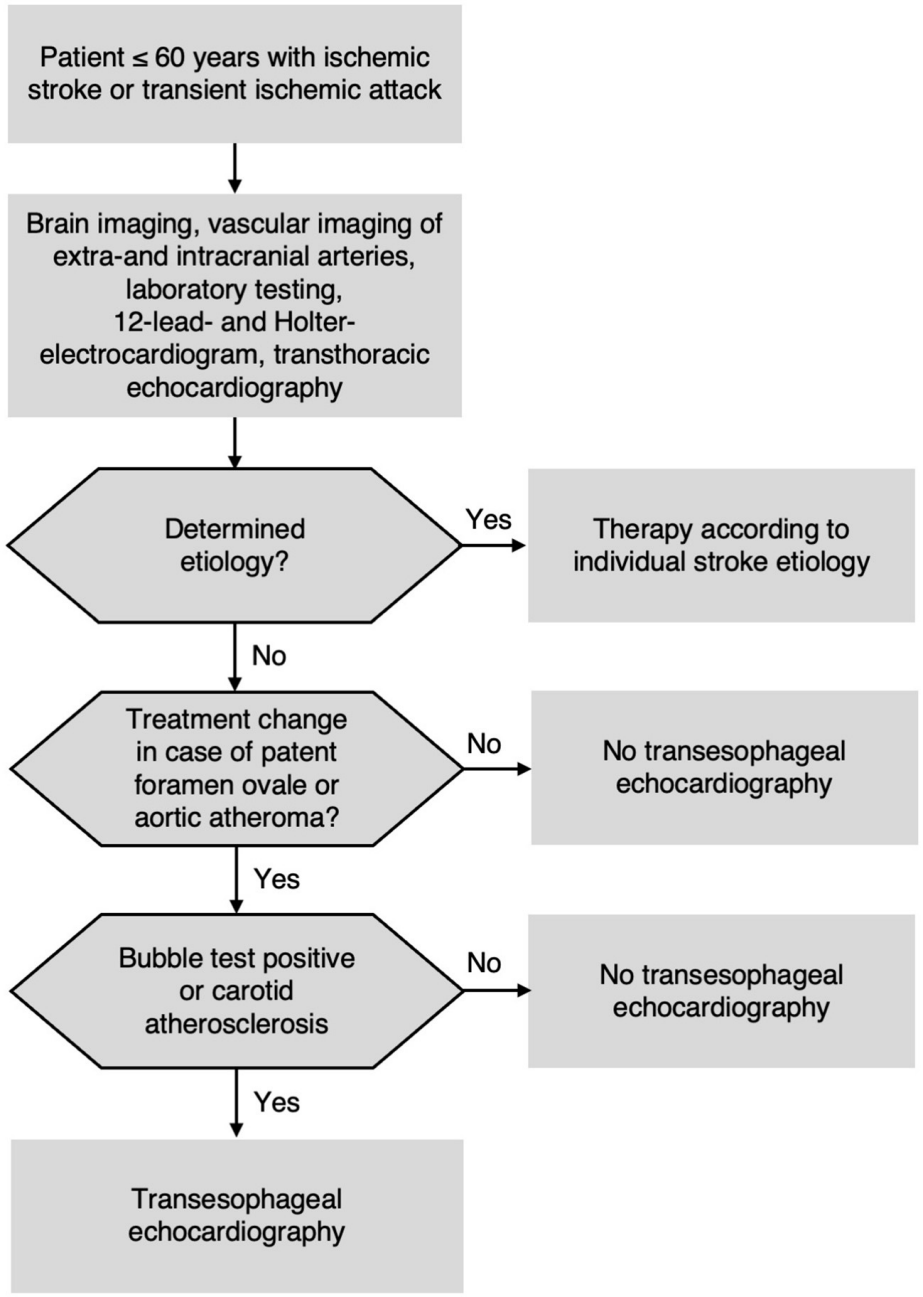
Proposed diagnostic algorithm for patients ≤60 years with acute ischemic stroke or transient ischemic attack (TIA).

Due to the overall TEE results and the high NPV of non-invasive ultrasound for the exclusion of therapy-relevant findings in TEE, we propose to establish a diagnostic algorithm to decide for/against TEE in patients ≤60 years with cryptogenic stroke after comprehensive routine diagnostics (Figure 3).

Our study confirms results from previous investigations assessing the incidence of TEE findings in patients with ischemic stroke of all ages (7, 8, 45). In particular, the most recent German multi-center study, overlapping with our study period, found virtually no TEE findings apart from PFO and aortic plaques in younger patients (9). There is plenty of evidence for using TCD bubble test as screening approach for PFO (18, 19, 28), and inferring presence of aortic atheroma from carotid IMT has been shown (23, 26). However, the aim of our study was to combine these two approaches and to test them prospectively in a well-defined young cryptogenic stroke population. We included 240 consecutive patients and performed standardized and strictly blinded diagnostics. As a result, we recommend a safe and pragmatic diagnostic algorithm (Figure 3) for the future performance of TEE in young patients. This approach could be beneficial for a large number of patients given that 25% of all stroke patients are younger than 60 years (46). In our experience, despite sparse evidence, TEE is often performed in young patients with stroke. Based on our findings, we are convinced that TEE could be omitted safely by applying our diagnostic algorithm (Figure 3) in this stroke subgroup.

Our study must be interpreted in the context of its design. First, only awake, cooperative, and non-aphasic patients can sufficiently perform Valsalva maneuver that is required for a high accuracy of the bubble test. Thus, our approach is not an option for patients in poor clinical condition. On the other hand, TEE was unsuccessful or prematurely aborted in 13 patients (4.7%) because of a physical barrier for the TEE probe, excessive choking, or other reasons. In such cases, our approach is a valuable alternative to TEE. Second, we failed to identify a small number of PFO, one ASD, and one complex aortic plaque using non-invasive ultrasound. None of them bore high risk of recurrent stroke or has well-established therapeutic consequence. Patent foramen ovale closure has an overall low risk reduction for recurrent stroke and might be more beneficial if the PFO is associated with high-risk features, such as large RLS and/or atrial septal aneurysm (10–12), hence it is not recommended for patients with only small RLS (14). Furthermore, the missed aortic plaque did not contain superimposed thrombi and thus had no high risk for embolism. Therefore, in our opinion the benefit resulting from TEE performance in these patients was limited. Finally, the non-invasive ultrasound was performed by a very experienced ultrasound team. Thus, confirmation of our results by different staff in other stroke cohorts would be beneficial to confirm the validity of our suggested diagnostic algorithm.

## Conclusions

Based on our findings, we suggest a pragmatic algorithm for the indication of TEE in patients ≤60 years of age with cryptogenic stroke or TIA. If PFO or aortic atheroma are relevant for further management, patients should undergo TCD bubble test and carotid ultrasound. If both are unremarkable, TEE can be omitted with a very low risk. Patients with positive bubble test or carotid atherosclerosis, however, should undergo TEE. The confirmation of PFO and embolic pattern of brain infarction can lead to PFO closure (14, 47). For patients with complex aortic plaques, hopefully future randomized-controlled trials will generate evidence for optimal secondary prevention.

## Data Availability Statement

Investigators may obtain access to de-identified patient data upon reasonable request to the corresponding author.

## Ethics Statement

The study was approved by the Ethics Committee of the University of Freiburg (approval number 259/19) before the inclusion of the first patient. All participants or their legally authorized representatives gave written informed consent prior to study inclusion.

## Author Contributions

EM, BG, CS, and DK screened and recruited patients and obtained patient data. AA performed TEE examinations. SG supervised TEE examinations and reviewed their findings. EM consolidated and analyzed the data and drafted the manuscript. AH and CS designed the study. EM, DK, BG, CDA, AA, SG, CS, and AH interpreted the data and revised the manuscript. All authors read and approved the final version of the manuscript.

## Funding

This article processing charge was funded by the Baden-Württemberg Ministry of Science, Research and Art, and the University of Freiburg in the funding program Open Access Publishing.

## Conflict of Interest

The authors declare that the research was conducted in the absence of any commercial or financial relationships that could be construed as a potential conflict of interest.

## Publisher's Note

All claims expressed in this article are solely those of the authors and do not necessarily represent those of their affiliated organizations, or those of the publisher, the editors and the reviewers. Any product that may be evaluated in this article, or claim that may be made by its manufacturer, is not guaranteed or endorsed by the publisher.
