# Supplementary material for: Bubble Test and Carotid Ultrasound to Guide Indication of Transesophageal Echocardiography in Young Patients With Stroke
Source: Front Neurol. 2022 Mar 4;13:836609. doi: 10.3389/fneur.2022.836609 (PMC8931264; doi:10.3389/fneur.2022.836609)
Supplement: Supplementary file 1 [file Table_1.docx]

Supplementary Material

# Supplementary Methods

## Screening for Patent Foramen Ovale (PFO)

For non-invasive PFO detection or exclusion, we performed a transcranial Doppler (TCD) bubble test in all patients, once at rest and once while performing an active Valsalva maneuver. We used a modified approach of the examination consensus^1^ to improve the test sensitivity by adding 1 ml of fresh patient blood to the agitated solution as suggested earlier^2,3^. First, the right middle cerebral artery was identified with transcranial 2D color-coded duplex sonography and the Doppler velocity spectrum was measured using pulse-waved Doppler ultrasound. Patients were trained to undertake an efficient Valsalva maneuver which was ruled sufficient in case of significant decrease in the Doppler velocity spectrum^4^. An agitated microbubble solution consisting of 8 ml of sterile saline, 1 ml of fresh patient blood and 1 ml of air was injected in the right cubital vein. If the right cubical vein was not accessible, a nearby vein of the right arm or, in a small number of cases, the left cubital vein was used alternatively. The first trial was carried out at rest, and after a break of at least one minute, a second trial was carried out following a Valsalva maneuver for a maximum of ten seconds that started five seconds after injection. Quantification of Right-to-Left Shunt (RLS) was based on the visual and audible detection of micro-embolic high-intensity signals (HITS) on the Doppler blood flow-velocity spectrum of the right middle cerebral artery within 14 seconds after injection and divided into four categories: none (no HITS), small (1-9 HITS), moderate (10 or more HITS), and large (“curtain effect”: embolic shower without discernible number of HITS). Bubble test was considered positive if there was one HITS or more either at rest or under Valsalva maneuver (see Figure 1).

## Ascertainment of cardiovascular risk factors

Overweight and obesity were classified according to the World Health Organization definition by calculating the body mass index (BMI)^5^. Due to the frequent temporary hypertension in acute stroke, hypertension was only considered as a risk factor if present and treated before stroke or newly diagnosed with other distinct signs of chronic hypertension (e.g., hypertrophic left ventricle, cerebral white matter disease). Diabetes was defined as known diabetes or presence of one of the following: HbA_1c_ >6.5%, fasting blood glucose >6.9 mmol/l (125 mg/dl), or anytime blood glucose >11.1 mmol/l (200mg/dl)^6^. Dyslipidemia was defined as one of: fasting low-density lipoprotein (LDL) >4.14 mmol/l (160 mg/dl), high-density lipoprotein (HDL) <1.03 mmol/l (40 mg/dl), triglycerides >1.70 mmol/l (150 mg/dl), or total cholesterol >5.17 mmol/l (200 mg/dl)^7^. Positive family history was defined as stroke or myocardial infarction before 55 years of age in father or in other male first degree relative, or before 65 years of age in mother of other female first degree relative^7^. Smoking and prevalence of cardiovascular and peripheral artery disease were obtained from patients’ history and charts.

# Supplementary Results

We prospectively screened 368 consecutive patients and included 276 patients. During screening, we excluded 92 patients for the following reasons: eligibility criteria not met (n = 36), refused consent (n = 32), bubble test not feasible (n = 11), missed by the study team (n = 11), blinding of the non-invasive ultrasound examiner not possible (n = 2).

Upon completion of the study, 27 patients were excluded because of incomplete non-invasive ultrasound or TEE data. TCD bubble test was incomplete in eight patients: Valsalva maneuver was insufficient because of aphasia or language barrier (n = 6), abortion of the test by the patient (n = 2). TEE data was missing in 19 patients: procedure unsuccessful (n = 7) or prematurely aborted (n = 6) because of physical barrier, excessive choking, agitation, or hypoxemia due to sedation; or not performed because of withdrawal of consent (n = 2), thrombocytopenia (n = 2), or novel medical findings in the meantime (n = 2). We excluded 9 further patients who underwent stroke diagnostics but had a non-ischemic diagnosis at discharge (migraine [n = 4], psychogenic symptoms [n = 2], brain tumor [n = 1], multiple sclerosis [n = 1], headache during sexual activity with focal neurological deficit [n = 1]). None of the patients had a diagnosis of carotid web.

We performed sensitivity analyses in the subset of patients with cryptogenic stroke only (n=163, 67.9%), of which 41 (25.1%) had negative non-invasive ultrasound. In those patients, combined non-invasive ultrasound reached a sensitivity of 98.6% (95% CI [92.3 100.0]) and an NPV of 97.6% (95% CI [87.1, 99.9]) for therapy-relevant findings in TEE. Specificity was 43.0% (95% CI [32.8, 53.7]) and PPV was 56.6% (95% CI [47.3, 65.5]). The positive likelihood ratio was 1.73 (95% CI [1.5, 2.1]) and the negative likelihood ratio was 0.03 (95% CI [0.00, 0.24]). TCD bubble test alone had a sensitivity of 91.0% (95% CI [81.5, 96.6]) and an NPV of 92.9% (95% CI [85.3, 97.4]) for detecting and excluding PFO in TEE. Specificity was 82.3% (95% CI [73.2, 89.3]) and PPV was 78.2% (95% CI [67.4, 86.8]). Carotid atherosclerosis alone showed a sensitivity of 100.0% (95% CI [54.1, 100.0]) and an NPV of 100.0% (95% CI [95.9, 100.0]) for the detection and exclusion of complex aortic plaques. Due to the overall low prevalence of complex aortic plaques (n = 10, 4.2%), specificity was 56.7% (95% CI [48.6, 64.6]) and PPV was only 8.1% (95% CI [3.0, 16.8]).

# Supplementary Tables

| **Characteristic** | **Value** |
| --- | --- |
| **Screening for Carotid Atherosclerosis** | |
| Carotid plaques overall – n (%) | 92 (38.3%) |
| Plaques left ICA – n (%) | 64 (26.7%) |
| Plaques right ICA – n (%) | 59 (24.6%) |
| Plaques left ECA – n (%) | 31 (12.9%) |
| Plaques right ECA – n (%) | 31 (12.9%) |
| Intima-Media-Thickness left CCA – median (IQR) mm | 0.60 (0.51-0.77) |
| Intima-Media-Thickness right CCA – median (IQR) mm | 0.59 (0.50-0.70) |
| Intima-Media-Thickness mean l/r CCA – median (IQR) mm | 0.60 (0.51-0.73) |
| **Screening for Patent Foramen Ovale** | |
| RLS at rest: None – n (%) | 160 (66.7%) |
| RLS at rest: Small – n (%) | 37 (15.4%) |
| RLS at rest: Moderate – n (%) | 37 (15.4%) |
| RLS at rest: Large – n (%) | 6 (2.5%) |
| RLS under Valsalva: None – n (%) | 123 (51.2%) |
| RLS under Valsalva: Small – n (%) | 36 (15.0%) |
| RLS under Valsalva: Moderate – n (%) | 34 (14.2%) |
| RLS under Valsalva: Large – n (%) | 40 (16.7%) |

Table S1. Results of the non-invasive ultrasound diagnostics of the 240 study participants.
ICA, internal carotid artery; ECA, external carotid artery; CCA, common carotid artery; RLS, right-to-left-shunt; IQR, interquartile range

| **Patients  n (%)** | **TEE findings** | **Further work-up** | **Therapeutic consequence** | | |
| --- | --- | --- | --- | --- | --- |
| **Non-invasive ultrasound negative (n = 68, 28.3%)** | | | |  |  |
| 1 (0.4%) | Contrast agent flow from pulmonary veins suspicious for PAVM Small PFO with small shunt at rest | Chest CT with contrast: no PAVM, but incidental bilateral pulmonary embolism | Oral anticoagulation and PFO occlusion | |  |
| 1 (0.4%) | PFO with small shunt at rest and moderate shunt under Valsalva maneuver | MRI without ischemic lesions | None | |  |
| 1 (0.4%) | Small PFO without shunt at rest and minimal shunt under Valsalva maneuver (two bubbles) | MRI with suspicion of punctiform deep ischemic lesion in pons | None | |  |
| 1 (0.4%) | Ostium secundum atrial septal defect without RLS | MRI without ischemic lesions | None | |  |
| 1 (0.4%) | LAA flow velocity 25 cm/s, spontaneous echo contrast without thrombi | ECG over five days without evidence of atrial fibrillation | None | |  |
| 1 (0.4%) | Atherosclerotic plaques in aortic arch with thickness up to 4.9mm (= complex plaque) | MRI without ischemic lesions | Individual recommendation for dual platelet inhibition for three months | |  |
| 2 (0.8%) | Contrast agent flow over pulmonary veins suspicious for PAVM | Chest CT with contrast: no PAVM | None | |  |
| **Non-invasive ultrasound positive (n = 172, 71.7%)** | | | |  |  |
| 93 (38.8%) | PFO with RLS Large n = 54 (58%) Moderate n = 27 (29%) Small n = 12 (13%) | Venous duplex imaging in 54 cases (55.6%)  Deep venous thrombosis in  six cases (6.5%) | PFO occlusion in 52 cases (55.9%) | | |
| 9 (3.8%) | Complex aortic plaques, median 6, IQR 2.3 mm thick; one with mobile thrombi |  | Individual recommendation for dual platelet inhibition for three months in n = 5 cases (55.6%) | | |
| 1 (0.4%) | LAA flow velocity 29.5 cm/s | ECG over four days without evidence of atrial fibrillation | None | | |
| 1 (0.4%) | small floating structure on the aortic valve of undetermined significance | Stable finding in follow-up TEE after eight months | None | | |

Table S2. TEE findings in patients with negative and positive non-invasive ultrasound.
Abbreviations: PAVM, pulmonary arteriovenous malformation; PFO, patent foramen ovale; LAA, left atrial appendage; TEE, transesophageal echocardiography; RLS, right-to-left-shunt

# Supplementary References

1. Jauss M, Zanette E. Detection of right-to-left shunt with ultrasound contrast agent and transcranial Doppler sonography. *Cerebrovasc Dis*. 2000;10:490-496. doi: 10.1159/000016119

2. Gentile M, De Vito A, Azzini C, Tamborino C, Casetta I. Adding blood to agitated saline significantly improves detection of right-to-left shunt by contrast-transcranial color-coded duplex sonography. *Ultrasound Med Biol*. 2014;40:2637-2641. doi: 10.1016/j.ultrasmedbio.2014.06.017

3. Shariat A, Yaghoubi E, Nemati R, Aghasadeghi K, Borhani Haghighi A. Comparison of agitated saline mixed with blood to agitated saline alone in detecting right-to-left shunt during contrast- transcranial Doppler sonography examination. *Acta Neurol Taiwan*. 2011;20:182-187. doi:

4. Tiecks FP, Lam AM, Matta BF, Strebel S, Douville C, Newell DW. Effects of the valsalva maneuver on cerebral circulation in healthy adults. A transcranial Doppler Study. *Stroke*. 1995;26:1386-1392. doi: 10.1161/01.str.26.8.1386

5. World Health Organization. Obesity: https://www.who.int/health-topics/obesity, Accessed 06/17/2021. WHO. 2021.

6. American Diabetes Association. 2. Classification and Diagnosis of Diabetes: Standards of Medical Care in Diabetes-2020. *Diabetes Care*. 2020;43:S14-S31. doi: 10.2337/dc20-S002

7. National Cholesterol Education Program Expert Panel on Detection E, Treatment of High Blood Cholesterol in A. Third Report of the National Cholesterol Education Program (NCEP) Expert Panel on Detection, Evaluation, and Treatment of High Blood Cholesterol in Adults (Adult Treatment Panel III) final report. *Circulation*. 2002;106:3143-3421. doi:
